# Aging is associated with reduced inflammatory disease activity independent of disease duration in relapsing multiple sclerosis trial populations

**DOI:** 10.1177/13524585241272938

**Published:** 2024-09-08

**Authors:** Eline ME Coerver, Sezgi Kaçar, Olga Ciccarelli, Maria P Sormani, Frederik Barkhof, Douglas L Arnold, Menno M Schoonheim, Zoé LE Van Kempen, Jop Mostert, Marcus W Koch, Joep Killestein, Arman Eshaghi, Bernard MJ Uitdehaag, Eva MM Strijbis

**Affiliations:** MS Center Amsterdam, Neurology, Vrije Universiteit Amsterdam, Amsterdam Neuroscience, Amsterdam UMC, Location VU Medical Centre, Amsterdam, The Netherlands; MS Center Amsterdam, Neurology, Vrije Universiteit Amsterdam, Amsterdam Neuroscience, Amsterdam UMC, Location VU Medical Centre, Amsterdam, The Netherlands; Queen Square Multiple Sclerosis Centre, Department of Neuroinflammation, UCL Queen Square Institute of Neurology, Faculty of Brain Sciences, University College London, London, UK; National Institute for Health and Care Research (NIHR) University College London Hospitals (UCLH) Biomedical Research Centre, London, UK; Department of Health Sciences (DISSAL), University of Genova, Genova, Italy; Ospedale Policlinico San Martino IRCCS, Genoa, Italy; Queen Square Multiple Sclerosis Centre, Department of Neuroinflammation, UCL Queen Square Institute of Neurology, Faculty of Brain Sciences, University College London, London, UK; Queen Square Institute of Neurology and Centre for Medical Image Computing, University College London, London, UK; MS Center Amsterdam, Department of Radiology and Nuclear Medicine, Vrije Universiteit Amsterdam, Amsterdam Neuroscience, Amsterdam UMC, Location VU medical center, Amsterdam, The Netherlands; Queen Square Multiple Sclerosis Centre, Department of Neuroinflammation, UCL Queen Square Institute of Neurology, Faculty of Brain Sciences, University College London, London, UK; NeuroRx Research and Montreal Neurological Institute, McGill University, Montreal, QC, Canada; Montreal Neurological Institute, McConnell Brain Imaging Centre, McGill University, Montreal, QC, Canada; MS Center Amsterdam, Anatomy and Neuroscience, Vrije Universiteit Amsterdam, Amsterdam Neuroscience, Amsterdam UMC, Location VU medical center, Amsterdam, The Netherlands; MS Center Amsterdam, Neurology, Vrije Universiteit Amsterdam, Amsterdam Neuroscience, Amsterdam UMC, Location VU Medical Centre, Amsterdam, The Netherlands; Department of Neurology, Rijnstate Hospital, Arnhem, The Netherlands; Department of Clinical Neurosciences, University of Calgary, Calgary, AB, Canada; MS Center Amsterdam, Neurology, Vrije Universiteit Amsterdam, Amsterdam Neuroscience, Amsterdam UMC, Location VU Medical Centre, Amsterdam, The Netherlands; Queen Square Multiple Sclerosis Centre, Department of Neuroinflammation, UCL Queen Square Institute of Neurology, Faculty of Brain Sciences, University College London, London, UK; Queen Square Institute of Neurology and Centre for Medical Image Computing, University College London, London, UK; MS Center Amsterdam, Neurology, Vrije Universiteit Amsterdam, Amsterdam Neuroscience, Amsterdam UMC, Location VU Medical Centre, Amsterdam, The Netherlands; MS Center Amsterdam, Neurology, Vrije Universiteit Amsterdam, Amsterdam Neuroscience, Amsterdam UMC, Location VU Medical Centre, Amsterdam, The Netherlands

**Keywords:** Aging, inflammation, relapsing MS

## Abstract

**Background:**

Higher age is associated with less inflammatory disease activity in relapsing-remitting multiple sclerosis (RRMS). It is unknown whether age itself or disease duration underlies this association.

**Objectives:**

This study investigated the effects of age, disease duration, and inflammatory disease activity in people with RRMS.

**Methods:**

Individual patient-level data from five large phase III randomized controlled trials (RCTs) was utilized to investigate the association of both age and disease duration with annualized relapse rate (ARR), contrast-enhancing lesions (CELs), and new T2 lesions on magnetic resonance imaging (MRI) at baseline and follow-up.

**Results:**

The data set included 5626 participants. Higher age was associated with lower ARRs, lower CEL number on MRI at baseline and follow-up, and lower new T2 lesion numbers at follow-up. This effect was present in all disease duration groups. For example, we found a lower number of new T2 lesions on MRI during follow-up in higher age groups compared to lower age groups, independent of disease duration.

**Conclusion:**

Aging in RRMS is associated with a lower risk of inflammatory disease activity, across different disease durations. Age should be taken into account when designing clinical trials and future research should investigate how age should be integrated into personalized predictions of treatment response and risk profiling.

## Introduction

Focal inflammatory disease activity in the central nervous system (CNS) that manifests as clinical relapses, new T2 lesions, and contrast-enhancing lesions (CELs) on magnetic resonance imaging (MRI) is the most prominent characteristic of relapsing-remitting multiple sclerosis (RRMS). Previous studies have shown that this focal inflammation decreases as people with RRMS age: higher age is associated with lower relapse rates and less focal inflammatory disease activity on MRI.^1–4^ In addition to this natural decrease of focal inflammation with advancing age, post hoc analyses of clinical trial data in MS also indicate that the relative efficacy of disease-modifying therapy (DMT) is lower in the higher age groups, as DMTs are mostly aimed at preventing focal inflammatory disease activity.^5–7^ Conversely, similar post hoc studies suggest that a relative similar efficacy of DMT was reported between older and younger patients.^8–10^

Although it has become increasingly clear from imaging studies that focal inflammation decreases in older patients and over time, it is not immediately apparent whether this decrease is caused by age itself, or rather, whether a longer disease duration may affect changes in the pathophysiology and decrease focal inflammation. Since age and disease duration are necessarily correlated, large data sets are required to answer this question.

Pooling data from phase III randomized controlled trials (RCTs) in RRMS allow us to investigate this question in a large and well-characterized data source to further investigate the association between age and focal inflammatory disease activity and investigate whether this association is present independent of disease duration. In this study, we investigated the association between age, disease duration, and focal inflammatory disease activity, as represented by clinical relapses, CELs, and new T2 lesions on MRI, using such a unique combined data set from five phase III clinical trials in RRMS.^11–14^

## Methods

### Study design and individual trial data sets

We performed an individual patient-level post hoc analysis of five completed phase III clinical trials in RRMS: OPERA I, OPERA II, BRAVO, CONFIRM, and DEFINE under the auspices of the International Progressive Multiple Sclerosis Alliance. The complete study protocols are described in detail in the original publications.^11–14^

#### OPERA I and OPERA II

The OPERA I and II trials investigated ocrelizumab treatment (600 mg every 24 weeks) in comparison to interferon beta-1a (IFNβ-1a) (44 μg three times weekly) treatment or placebo. People with relapsing MS aged between 18 and 55 years and an Expanded Disability Status Scale (EDSS) between 0 and 5.5 were included. Participants had to have had at least two documented clinical relapses within the previous 2 years or one clinical relapse in the year before screening. The OPERA I and OPERA II study participants were recruited from 141 and 166 sites over 32 and 24 countries, respectively.^11^

#### BRAVO

The BRAVO trial studied the efficacy and safety of laquinimod (0.6 mg once daily) compared to IFNβ-1a (30 μg once weekly) treatment. People with RRMS aged between 18 and 56 years and with an EDSS between 0 and 5.5 were included. Participants had to have had at least one clinical relapse in the previous year, two relapses in the previous 2 years, or one in the previous 1–2 years plus 1 or more CELs in the previous year. Participants were recruited from 155 sites from 18 countries, and assessment was done in a rater-blinded design.^12^

#### DEFINE

The DEFINE trial studied the efficacy and safety of oral BG-12 (dimethyl fumarate) at a dose of 240 mg twice daily and BG-12 at a dose of 240 mg three times daily compared to placebo. People with RRMS aged between 18 and 55 years and with an EDSS of 0–5.5 were included. At least one clinically documented relapse in the year before inclusion or one CEL on MRI between 0 and 6 weeks before randomization had to be present. Subjects of the DEFINE study were recruited from 198 sites over 28 countries.^13^

#### CONFIRM

The CONFIRM trial studied the efficacy and safety of oral BG-12 at a dose of 240 mg two or three times daily in comparison to an active reference medication (glatiramer acetate (20 mg once daily)) or placebo. People with RRMS aged between 18 and 55 years and with an EDSS between 0 and 5.5 were included. At least one clinically documented relapse in the previous year or at least one CEL 0–6 weeks before randomization had to be present. Subjects of the CONFIRM study were recruited from 200 sites over 28 countries.^14^

### Data collection and outcome measures

We used standard clinical and MRI parameters at baseline and during the follow-up period of the trials. In addition, we collected the number of relapses before inclusion and between baseline and follow-up. Clinical parameters at baseline included the age of the participant, date of MS onset, date of diagnosis, and EDSS. MRI parameters include the number of CELs and number of new T2 lesions at baseline and during follow-up. We calculated the annualized relapse rate (ARR) during follow-up by dividing the number of relapses by the participant-years of follow-up. We divided the number of CELs and number of T2 lesions during follow-up into the following categories: “0,” “1–2,” “3–4,” or “5 or more” lesions. We divided participant age into the following six groups, based on bins of 5 years at baseline: “18–24 years,” “25–30 years,” “31–35 years,” “36–40 years,” “41–45 years,” “46–50 years,” and “51–56 years.”

To investigate whether disease duration is an important factor in the association between age and focal inflammation, we subdivided the cohort into three groups with different disease durations. To obtain a proportional number of participants in each subgroup, the subgroups we used were “0–4 years,” “5–9 years,” and “10 or more years.” Disease duration was defined as the time from the onset date (i.e. date of first symptoms) to the start date of the trial.

### Statistical analysis

We investigated the association between age group and outcome measures (ARR, number of CELs, number of T2 lesions) with negative binomial mixed model analyses, in which study site and site ID were added as random factors. Only participants with a minimum follow-up of 6 months were included in the ARR calculation during the trial. The age group of “25–30 years” was used as the reference group, as the lowest age group (“18–24 years”) was relatively small, especially in the 5–9 and ⩾10 years disease duration subgroups. We repeated all analyses with medication arm also included as random factor, using three groups for the medication arm: (1) a placebo group, (2) a first-line medication arm, and (3) a second-line medication arm. The first-line medication arm included participants with the following intervention: IFNβ-1a (Avonex or Rebif), “dimethyl fumarate (twice or three times a day), glatiramer acetate, or laquinimod. The second-line medication arm includes participants who obtained the intervention with ocrelizumab. In addition, we repeated the analyses for each disease duration group separately. The significance level for all statistical analyses was set at *p* < 0.05. All statistical analyses were performed with the R statistical software package for Windows version 4.0.3.^15^ We repeated all analyses with age as a continuous factor using linear mixed models.

### Standard protocol approvals, registrations, and patient consent

Data from the BRAVO, CONFIRM, DEFINE, and OPERA I and II trials are provided by the International Progressive MS Alliance (IPMSA) with written informed consent. The Institutional Review Board at the Montreal Neurological Institute (MNI), Quebec, Canada, approved this study (reference no. IRB00010120). All participants provided written informed consent prior to participation in study protocols.^11–14^ The data presented in this study are available upon reasonable request from the corresponding author.

## Results

### Baseline characteristics

In total, 5650 participants were included in the combined data set. The data set of the BRAVO trial included 1348 participants, CONFIRM 1421 participants, DEFINE 1233 participants, OPERA I 815 participants, and OPERA II 833 participants. Baseline characteristics per trial and per treatment arm are shown in Table 1. In addition, baseline characteristics per age group are shown in Table 2. As expected, disease duration was higher in participants in the older age groups: for example, the mean disease duration for the “18–24 years” age group was 2.87 years (standard deviation (SD) = 2.65) compared to 9.71 years (SD = 8.43) for the “51–56” age group. EDSS scores were also significantly higher in the older age groups compared to the reference “25–30” age group (Table 2).

**Table 1.** Baseline characteristics per trial and medication arm.

	TOTAL	OPERA I		OPERA II		BRAVO			CONFIRM				DEFINE		
		OCR	IFNβ-1a	OCR	IFNβ-1a	Laquinimod	IFNβ-1a	Placebo	BG-12 (BID)	BG-12 (TID)	GA	Placebo	BG-12 (BID)	BG-12 (TID)	Placebo
Number of included participants	5650	815		833		1348			1421				1233		
		408	407	415	418	436	450	462	361	345	353	362	409	416	408
Age, mean (SD)	37.3 (9.25)	37 (9.33)	36.93 (9.34)	37.13 (9.10)	37.35 (8.97)	36.89 (9.33)	38.22 (9.46)	37.21 (9.58)	37.24 (9.39)	37.32 (9.38)	36.27 (9.05)	36.43 (9.24)	37.60 (9.10)	38.31 (8.90)	38.00 (9.16)
Age group, *n* (%)															
18–24 years	621 (11.0)	54 (13.2)	53 (13.0)	44 (10.6)	47 (11.2)	53 (12.2)	42 (9.3)	52 (11.3)	37 (10.2)	41 (11.9)	39 (11.0)	43 (11.9)	38 (9.3)	34 (8.2)	44 (10.8)
25–30 years	872 (15.4)	55 (13.5)	61 (15.0)	66 (15.9)	54 (12.9)	67 (15.4)	60 (13.3)	71 (15.4)	65 (18.0)	54 (15.7)	76 (21.5)	70 (19.3)	70 (17.1)	58 (13.9)	45 (11.0)
31–35 years	851 (15.1)	61 (15.0)	68 (16.7)	73 (17.6)	75 (17.9)	68 (15.6)	65 (14.4)	77 (16.7)	48 (13.3)	45 (13.0)	46 (13.0)	54 (14.9)	58 (14.2)	57 (13.7)	56 (13.7)
36–40 years	1171 (20.7)	91 (22.3)	76 (18.7)	87 (21.0)	88 (21.1)	82 (18.8)	100 (22.2)	77 (16.7)	74 (20.5)	76 (22.0)	75 (21.2)	71 (19.6)	89 (21.8)	90 (21.6)	95 (23.3)
41–45 years	812 (14.4)	65 (15.9)	60 (14.7)	53 (12.8)	69 (16.5)	66 (15.1)	62 (13.8)	68 (14.7)	49 (13.6)	40 (11.6)	48 (13.6)	44 (12.2)	53 (13.0)	70 (16.8)	65 (15.9)
46–50 years	854 (15.1)	47 (11.5)	56 (13.8)	59 (14.2)	50 (12.0)	59 (13.5)	60 (13.3)	63 (13.6)	58 (16.1)	63 (18.3)	49 (13.9)	64 (17.7)	72 (17.6)	78 (18.8)	76 (18.6)
51–56 years	469 (8.3)	35 (8.6)	33 (8.1)	33 (8.0)	35 (8.4)	41 (9.4)	61 (13.6)	54 (11.7)	30 (8.3)	26 (7.5)	20 (5.7)	16 (4.4)	29 (7.1)	29 (7.0)	27 (6.6)
Female sex, *n* (%)	3903 (69.1)	269 (65.9)	269 (66.1)	269 (64.8)	280 (67.0)	282 (64.7)	307 (68.2)	324 (70.1)	246 (68.1)	250 (72.5)	249 (70.5)	251 (69.3)	295 (72.1)	306 (73.6)	306 (75.0)
Disease duration, years, mean (SD)															
0–4 years	1.90 (1.27)	1.62 (1.31)	1.82 (1.29)	1.73 (1.37)	1.6 (1.29)	2.24 (1.12)	2.18 (1.18)	2.14 (1.15)	2.17 (1.19)	2.02 (1.22)	1.99 (1.16)	2.31 (1.22)	2.27 (1.24)	2.11 (1.21)	2.25 (1.20)
5–9 years	6.71 (1.44)	6.81 (1.41)	6.66 (1.49)	6.99 (1.39)	6.94 (1.49)	6.51 (1.38)	6.48 (1.42)	6.63 (1.45)	6.93 (1.45)	6.83 (1.43)	6.96 (1.41)	6.63 (1.40)	6.94 (1.31)	6.69 (1.40)	6.85 (1.38)
⩾10 years	15.2 (5.22)	15.4 (5.03)	15.2 (5.46)	15.3 (5.13)	14.8 (5.04)	14.8 (5.36)	14.7 (4.57)	16.1 (6.03)	16.0 (5.81)	15.9 (5.39)	14.7 (4.67)	15.1 (4.91)	15.4 (5.54)	15.3 (4.76)	16.2 (5.52)
EDSS, median (IQR)	2.5 (1.5–3.5)	2.5 (2.0–3.5)	2.5 (1.5–3.5)	2.5 (2.0–3.5)	2.5 (2.0–4.0)	2.5 (1.5–3.5)	2.5 (1.5–3.5)	2.5 (1.5–3.5)	2.5 (1.5–3.5)	2.5 (1.5–3.5)	2.5 (1.5–3.5)	2.5 (1.5–3.5)	2.0 (1.5–3.5)	2.0 (1.5–3.0)	2.5 (1.5–3.5)
T25FW, seconds, mean (SD)	7.15 (8.45)	8.04 (9.02)	6.99 (6.49)	7.82 (10.79)	7.49 (11.24)	6.07 (2.95)	6.33 (5.34)	6.05 (3.06)	7.0 (6.64)	7.38 (8.13)	7.55 (8.81)	6.64 (4.57)	7.77 (12.0)	7.15 (8.69)	8.17 (12.92)
9-HPT, seconds, mean (SD)	23.36 (12.05)	24.95 (16.52)	23.33 (6.20)	23.97 (8.38)	24.63 (9.88)	22.49 (6.61)	22.46 (5.63)	23.14 (13.35)	22.9 (8.45)	22.7 (12.14)	22.61 (6.49)	23.47 (12.74)	23.84 (18.01)	22.91 (15.25)	23.81 (17.41)

Baseline characteristics for the overall data set, along with comprehensive breakdowns specific to each trial and medication arm. These baseline characteristics cover essential details including the total number of patients, age distribution, gender composition, disease duration, EDSS (Expanded Disability Status Scale) scores, T25FW (Timed 25-Foot Walk) results, and 9-HPT (9-Hole Peg Test) scores.

BG-12: dimethyl fumarate; BID: bis in die, twice a day; EDSS: Expanded Disability Status Scale; GA: glatiramer acetate; IFNβ-1a: interferon-beta-1a; OCR: ocrelizumab; TID: ter in die, three times a day; T25FW: timed 25-food walk test; 9-HPT: nine-hole peg test.

**Table 2.** Baseline characteristics per age group.

	18–24 *n* = 614	25–30 *n* = 870	31–35 *n* = 849	36–40 *n* = 1166	41–45 *n* = 812	46–50 *n* = 854	51–56 *n* = 469
Years since diagnosis, mean (SD)	1.81 (2.08)	2.85 (3.08)	3.85 (4.11)	4.49 (4.79)	4.90 (5.48)	6.14 (6.29)	6.01 (6.65)
Years since onset, mean (SD)	2.87 (2.65)	4.44 (3.69)	5.70 (4.60)	7.02 (5.83)	7.43 (6.33)	9.21 (7.25)	9.71 (8.43)
Number of T2 lesions, mean (SD)	55.19 (45.35)	51.51 (40.8)	54.91 (42.37)	52.38 (35.86)	45.38 (36.69)	42.79 (26.7)	49.04 (34.35)
Number of CELs, mean (SD)	3.42 (9.69)	2.26 (5.60)	2.18 (5.88)	1.63 (4.34)	1.06 (2.55)	0.98 (3.21)	0.73 (2.43)
ARR, 1 year before inclusion, mean (SD)	1.99 (3.60)	1.50 (2.87)	1.45 (2.67)	1.38 (2.54)	1.34 (2.59)	1.19 (2.15)	0.91 (2.21)
ARR, 2 years before inclusion, mean (SD)	0.86 (3.01)	0.57 (1.33)	0.45 (1.03)	0.40 (0.94)	0.37 (0.81)	0.38 (0.90)	0.23 (0.58)
EDSS, median (IQR)	2.0 (1.5–3.0)	2.0 (1.5–3.0)	2.5 (1.5–3.5)	2.5 (1.5–3.5)	2.5 (2.0–3.5)	3.0 (2.0–4.0)	3.5 (2.5–4.0)

Baseline characteristics with age-specific breakdowns for the entire data set. The baseline characteristics include information such as years since diagnosis, years since onset, the number of T2 lesions, the number of contrast-enhancing lesions (CELs), the number of relapses, annualized relapse rate (ARR), and EDSS (Expanded Disability Status Scale) scores.

ARR: annualized relapse rate; CELs = contrast-enhancing lesions; EDSS = Expanded Disability Status Scale.

### Disease activity at baseline

#### Age versus clinical disease activity

Average relapse count at baseline (i.e. in the year before inclusion) was lower in older age groups. For example, the ARR in the last year before inclusion in the “18–24 years” group was 1.99 (SD = 3.60) compared to 0.91 (SD = 2.21) for the “51–56 years” age group. The ARR in the 2 years before inclusion was also lower in older age groups: the ARR for the “18–24 years” group was 0.86 (SD = 3.01) compared to 0.23 (SD = 0.58) for the “51–56 years” age group (Tables 2 and 3). Higher age was also associated with a lower ARR in the 2 years before inclusion (e.g. 51–56 years vs 25–30 years (reference): β = −0.311, 95% confidence interval (CI) = −0.50 to −0.11) (Table 3). This finding was statistically significant when comparing the reference group to all other age groups, except for the “31–35 years” group. We found similar results when repeating the analyses with age as a continuous variable.

**Table 3.** Age versus disease activity at baseline.

		Number of CELs					ARR, 2 years before inclusion			
	*N*	Mean	RR	*p*	95% CI	*N*	Mean	β	*p*	95% CI
Age										
18–24 years	332	1.90	1.080	0.154	0.97 to 1.20	385	0.86	0.306	<0.001**	0.13 to 0.48
25–30 years	432	1.76		*Ref*		581	0.57		*Ref*	
31–35 years	479	1.68	0.955	0.363	0.87 to 1.05	517	0.45	−0.102	0.211	−0.26 to 0.06
36–40 years	595	1.60	0.908	0.049*	0.83 to 0.99	764	0.40	−0.174	0.019*	−0.32 to −0.03
41–45 years	442	1.46	0.829	<0.001***	0.75 to 0.92	506	0.37	−0.197	0.016*	−0.36 to −0.04
46–50 years	392	1.39	0.788	<0.001***	0.71 to 0.88	573	0.38	−0.216	0.007**	−0.37 to −0.06
51–56 years	291	1.32	0.751	<0.001***	0.66 to 0.85	259	0.23	−0.311	0.002**	−0.50 to −0.11

Results of regression analysis examining the relationship between age groups and disease duration activity at baseline. Rate ratios or beta coefficients, along with their corresponding 95% confidence intervals, illustrate the difference between each age group and the reference age group (25–30 years) for the number of contrast-enhancing lesions (CELs) and annualized relapse rate (ARR) across various disease duration subgroups.

ARR: annualized relapse rate; CELs: contrast-enhancing lesions; CI: confidence interval; RR: rate ratio; β: regression coefficient.

* *p* < 0.050; **p < 0.010; ***p < 0.001.

#### Age versus radiological disease activity

The number of CELs at baseline was significantly lower when comparing the reference group to the age groups of 36 years and older. Specifically, the average number of CELs in the “51–56 years” group was 0.73 (SD = 2.43), compared to 3.42 (SD = 9.69) in the “18–24 years” group (Table 2). Higher age was also significantly associated with a lower number of CELs on baseline MRI (51–56 years vs 25–30 years (reference): rate ratio (RR) = 0.751, 95% CI = 0.66 to 0.85) (Tables 2 and 3). This pattern was consistent for all age groups above 36 years. There were no significant differences in the number of T2 lesions on baseline MRI per age group (Table 2). The number of CELs on baseline MRI was lower in older participants (RR = 0.988; CI = 0.98 to 0.99; *p* < 0.005) when the analyses were repeated using age as a continuous variable.

#### Age versus disease activity, corrected for disease duration

The number of CELs at baseline decreased in the higher age groups in all subgroups of disease duration: in participants with a disease duration of 0–4 years (51–56 years vs 25–30 years (reference): RR = 0.801, 95% CI = 0.66–0.97), in the subgroup with a disease duration of 5–9 years (51–56 years vs 25–30 years (reference): RR = 0.775, 95% CI = 0.62–0.96), and in those with a disease duration of >10 years (51–56 years vs 25–30 years (reference): RR = 0.701, 95% CI = 0.55 to 0.90) (Table 4). This pattern was consistent from 36 years onward. We found similar results in all disease duration subgroups: the number of CELs and ARR were lower in the higher age groups across all disease duration subgroups when analyses were repeated using age as a continuous variable.

**Table 4.** Age versus disease duration activity at baseline per disease duration group.

		Disease duration 0–4 years								
		Number of CELs					ARR, 2 years before inclusion			
	*N*	Mean	RR	*p*	95% CI	*N*	Mean	β	*p*	95% CI
Age										
18−24 years	263	1.89	1.086	0.210	0.95 to 1.24	267	0.72	0.266	0.002*	0.01 to 0.43
25−30 years	238	1.75		*Ref*		313	0.43		*Ref*	
31−35 years	229	1.69	0.975	0.713	0.85 to 1.12	203	0.35	−0.040	0.657	−0.22 to 0.14
36−40 years	259	1.61	0.927	0.272	0.81 to 1.06	260	0.26	−0.152	0.071	−0.32 to 0.01
41−45 years	192	1.56	0.904	0.179	0.78 to 1.05	166	0.28	−0.115	0.237	−0.30 to 0.07
46−50 years	124	1.44	0.831	0.038*	0.70 to 0.99	154	0.40	−0.059	0.553	−0.25 to 0.13
51−56 years	98	1.39	0.801	0.024*	0.66 to 0.97	79	0.19	−0.195	0.126	−0.44 to 0.05
		Disease duration 5–9 years								
Age										
18−24 years	61	2.00	1.110	0.339	0.90 to 1.37	104	1.24	0.582	0.015*	0.11 to 1.05
25−30 years	149	1.81		*Ref*		191	0.68		*Ref*	
31−35 years	156	1.66	0.920	0.339	0.78 to 1.09	177	0.54	−0.128	0.533	−0.53 to 0.27
36−40 years	150	1.49	0.824	0.032*	0.69 to 0.98	237	0.41	−0.271	0.158	−0.64 to 0.10
41−45 years	109	1.44	0.799	0.025*	0.66 to 0.97	128	0.36	−0.290	0.198	−0.73 to 0.15
46−50 years	108	1.45	0.807	0.033*	0.66 to 0.98	132	0.34	−0.344	0.125	−0.78 to 0.09
51−56 years	81	1.40	0.775	0.023*	0.62 to 0.96	65	0.21	−0.449	0.113	−1.00 to −0.10
		Disease duration ⩾10 years								
Age										
18−24 years	8	1.62	0.952	0.867	0.53 to 1.70	12	0.80	−0.049	0.866	−0.02 to 0.13
25−30 years	45	1.71		*Ref*		77	0.86		*Ref*	
31−35 years	94	1.69	0.992	0.950	0.78 to 1.26	135	0.49	−0.350	0.009**	−0.10 to 0.04
36−40 years	186	1.68	0.983	0.876	0.79 to 1.22	264	0.52	−0.340	0.005**	−0.16 to −0.01
41−45 years	141	1.33	0.774	0.025*	0.62 to 0.97	208	0.45	−0.420	<0.001***	−0.14 to 0.02
46−50 years	160	1.29	0.751	0.015*	0.60 to 0.95	285	0.40	−0.480	<0.001***	−0.15 to 0.01
51−56 years	112	1.21	0.701	0.005*	0.55 to 0.90	115	0.27	−0.580	<0.001***	−0.23 to −0.04

Results of regression analysis examining the relationship between age groups and disease duration activity at baseline per disease duration group. Rate ratios or beta coefficients, along with their corresponding 95% confidence intervals, illustrate the difference between each age group and the reference age group (25–30 years) for the number of contrast-enhancing lesions (CELs) and annualized relapse rate (ARR) across various disease duration subgroups.

ARR: annualized relapse rate; CELs: contrast-enhancing lesions; CI: confidence interval; RR: rate ratio; β: regression coefficient.

* *p* < 0.050; **p < 0.010; ***p < 0.001.

### Disease activity during follow-up

#### Age versus clinical disease activity

**Figure 1.**
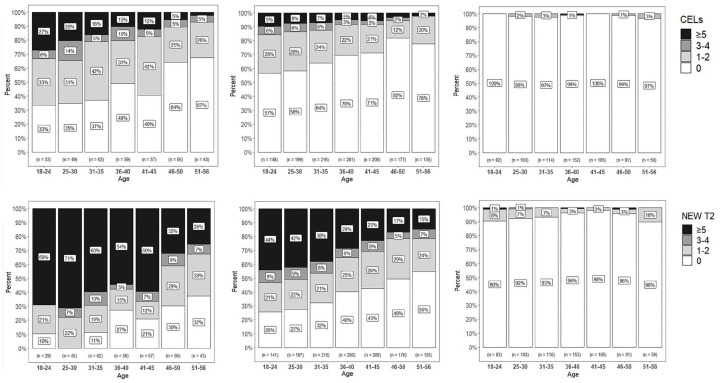
Age versus disease activity during follow-up for the different DMT categories. Proportion of trial participants with contrast-enhancing lesions (CELs) or new T2 lesions per age group for the different DMT categories in the different trials. In each of the panels, green means no CELs/new T2 lesions, orange means 1–2 CELs/new T2 lesions, purple means 3–4 CELs/new T2 lesions, and pink means 5 or more CELs/new T2 lesions. In the panels with “first-line DMT,” we included the trial arms with interferon-beta-1a, dimethyl fumarate (twice or three times a day), glatiramer acetate, and laquinimod. In the second-line DMT panel, we included the ocrelizumab arm of the opera trials. CELs: contrast-enhancing lesions; RR: rate ratio; β: regression coefficient.

The average ARR for the “18–24 years” age group was 0.38 (SD = 0.03) compared to 0.19 (SD = 0.04) for the “51–56 years” age group. This difference was still present when corrected for sex and treatment arm (51–56 years vs 25–30 years: β = −0.127, 95% CI = −0.20 to −0.06) (Table 5 and Figure 1) and was mostly statistically significant when comparing the reference group to the older age groups (starting from 36 years onward). The ARR during the trial period was also lower in older compared to younger participants (β = −0.005; CI = 0.01 to −0.00; *p*< 0.005) when analyses were repeated, and age was used as a continuous variable.

**Table 5.** Age versus disease activity during follow-up.

		Number of CELs						Number of new T2 lesions						ARR	
	*N*	Mean	RR	*p*	95% CI	*N*	Mean	RR	*p*	95% CI	*N*	Mean	β	*p*	95% CI
Age															
18–24 years	263	1.54	1.025	0.675	0.91 to 1.15	253	2.26	1.030	0.578	0.93 to 1.14	374	0.38	0.058	0.081	−0.01 to 0.12
25–30 years	351	1.53		*Ref*		345	2.30		*Ref*		505	0.33		*Ref*	
31–35 years	392	1.47	0.959	0.437	0.86 to 1.07	393	2.20	0.958	0.367	0.87 to 1.05	546	0.32	−0.002	0.937	−0.06 to 0.06
36–40 years	492	1.35	0.894	0.030*	0.81 to 0.99	492	1.94	0.857	<0.002**	0.78 to 0.94	721	0.25	−0.074	0.009**	−0.13 to −0.02
41–45 years	370	1.38	0.897	0.053	0.80 to 1.00	368	1.95	0.845	<0.002**	0.77 to 0.93	531	0.26	−0.059	0.052	−0.12 to 0.00
46–50 years	324	1.24	0.801	<0.001***	0.71 to 0.90	323	1.75	0.754	<0.001***	0.68 to 0.84	508	0.26	−0.066	0.033*	−0.13 to −0.01
51–56 years	237	1.24	0.784	<0.001***	0.69 to 0.89	237	1.71	0.709	<0.001***	0.63 to 0.79	318	0.19	−0.127	<0.001***	−0.20 to −0.06

Results of regression analysis examining the relationship between age groups and disease duration activity during follow-up. Rate ratios or beta coefficients, along with their corresponding 95% confidence intervals, illustrate the difference between each age group and the reference age group (25–30 years) for the number of contrast-enhancing lesions (CELs), number of new T2 lesions, and the annualized relapse rate (ARR).

ARR: annualized relapse rate; CELs: contrast-enhancing lesions; CI: confidence interval; RR: rate ratio; β: regression coefficient.

* *p* < 0.050; **p < 0.010; ***p < 0.001.

#### Age versus radiological disease activity

The number of CELs on follow-up MRI was lower in the higher age groups (51–56 years vs 25–30 years (reference): RR = 0.784, 95% CI = 0.69 to 0.89). The number of new T2 lesions was also lower in higher age groups (51–56 years vs 25–30 years (reference): RR = 0.703, 95% CI = 0.63 to 0.79). Both findings persisted from 36 years onward and were still present when correcting for sex and treatment arm (Table 5 and Figure 1). Similarly, the number of new on-trial T2 lesions was lower in older participants (RR = 0.987; 95% CI = 0.98 to 0.99; *p* = 0.000) when analysis was repeated with age as a continuous variable. The results were similar for the number of on-trial CELs (RR = 0.991; 95% CI = 0.98 to 0.99; *p* < 0.005).

#### Age versus disease activity, corrected for disease duration

**Figure 2.**
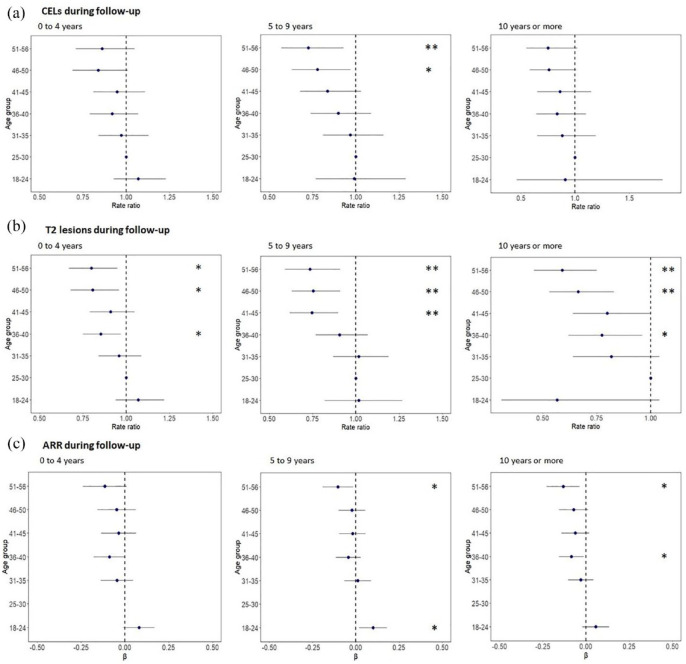
Age versus disease activity during follow-up per disease duration group. Rate ratio or beta coefficient and 95% confidence intervals show the difference between each of the age groups in relation to the reference age group of 25–30 years for the number of CELs, new T2 lesions, and ARR across the different disease duration subgroups. ARR: annualized relapse rate; CELs: contrast-enhancing lesions; β: regression coefficient. *p < 0.050; **p < 0.010; ***p < 0.001.

The number of new T2 lesions on MRI during follow-up decreased in the higher age groups in all disease duration subgroups: participants with a disease duration of 0–4 years (51–56 years vs 25–30 years (reference): RR = 0.799, 95% CI = 0.67 to 0.95), people with a disease duration of 5–9 years (51–56 years vs 25–30 years (reference): RR = 0.735, 95% CI = 0.59 to 0.91), and the group with a disease duration >10 years (51–56 years vs 25–30 years (reference): RR = 0.591, 95% CI = 0.46 to 0.75) (Table 6 and Figure 2). This pattern was observed across all age groups above 36 years. We also observed similar patterns across disease duration subgroups for CELs, new T2 lesions, and ARR when analyses were repeated with age as a continuous variable. Furthermore, when comparing participants with disease durations of 0–4 years to those of similar age with durations of 5–9 years and ⩾10 years, no significant differences were found in CEL, ARR, or new T2 lesion patterns among the various disease duration groups. Finally, no significant difference was found in the results when separately focusing on females or males.

**Table 6.** Age versus disease activity during follow-up per disease duration group.

			Disease duration 0–4 years												
			Number of CELs				Number of new T2 lesions					ARR			
	*N*	Mean	RR	*p*	95% CI	*N*	Mean	RR	*p*	95% CI	*N*	Mean	β	*p*	95% CI
Age															
18–24 years	211	1.55	1.070	0.355	0.93 to 1.23	202	2.29	1.070	0.294	0.94 to 1.21	293	0.38	0.081	0.077	−0.01 to 0.17
25–30 years	203	1.46		*Ref*		198	2.21		*Ref*		282	0.30		*Ref*	
31–35 years	189	1.42	0.972	0.706	0.84 to 1.13	187	2.11	0.960	0.539	0.84 to 1.09	251	0.24	−0.044	0.351	−0.14 to 0.05
36–40 years	219	1.33	0.919	0.265	0.79 to 1.07	218	1.89	0.854	0.016*	0.75 to 0.97	280	0.19	−0.088	0.058	−0.18 to 0.00
41–45 years	159	1.40	0.948	0.510	0.81 to 1.11	158	2.00	0.911	0.186	0.79 to 1.05	200	0.26	−0.035	0.494	−0.13 to 0.06
46–50 years	103	1.19	0.839	0.068	0.69 to 1.01	103	1.72	0.807	0.014*	0.68 to 0.96	147	0.24	−0.046	0.412	−0.15 to 0.06
51–56 years	80	1.27	0.862	0.140	0.71 to 1.05	80	1.82	0.799	0.010*	0.67 to 0.95	102	0.16	−0.114	0.073	−0.24 to 0.01
			Disease duration 5–9 years												
Age															
18–24 years	46	1.54	0.992	0.953	0.77 to 1.29	45	2.22	1.017	0.883	0.82 to 1.27	70	0.38	0.101	0.015*	0.02 to 0.18
25–30 years	116	1.65		*Ref*		115	2.40		*Ref*		165	0.28		*Ref*	
31–35 years	124	1.59	0.969	0.732	0.81 to 1.16	126	2.43	1.017	0.830	0.87 to 1.19	181	0.28	0.012	0.753	−0.06 to 0.09
36–40 years	118	1.47	0.899	0.267	0.74 to 1.09	119	2.18	0.906	0.237	0.77 to 1.07	196	0.23	−0.042	0.252	−0.11 to 0.03
41–45 years	92	1.37	0.838	0.099	0.68 to 1.03	92	1.77	0.747	0.002**	0.62 to 0.90	132	0.27	−0.018	0.640	−0.09 to 0.06
46–50 years	85	1.33	0.779	0.022*	0.63 to 0.97	84	1.89	0.755	0.003**	0.63 to 0.91	134	0.27	−0.022	0.585	−0.10 to 0.06
51–56 years	61	1.20	0.727	0.009**	0.57 to 0.93	61	1.75	0.735	0.005**	0.59 to 0.91	86	0.17	−0.103	0.023*	−0.19 to −0.01
			Disease duration ⩾10 years												
Age															
18–24 years	6	1.50	0.909	0.786	0.46 to 1.81	6	1.33	0.567	0.067	0.31 to 1.04	11	0.38	0.057	0.145	−0.02 to 0.13
25–30 years	32	1.56		*Ref*		32	2.44		*Ref*		58	0.32		*Ref*	
31–35 years	79	1.41	0.882	0.415	0.65 to 1.19	80	2.05	0.818	0.098	0.64 to 1.04	114	0.29	−0.029	0.435	−0.10 to 0.04
36–40 years	155	1.29	0.834	0.198	0.64 to 1.10	155	1.83	0.775	0.021*	0.62 to 0.96	245	0.22	−0.083	0.023*	−0.16 to −0.01
41–45 years	119	1.36	0.860	0.304	0.65 to 1.15	118	2.01	0.799	0.052	0.64 to 1.00	199	0.25	−0.061	0.135	−0.14 to 0.02
46–50 years	136	1.22	0.759	0.059	0.57 to 1.01	136	1.69	0.664	<0.001***	0.53 to 0.83	227	0.24	−0.072	0.093	−0.15 to 0.01
51–56 years	96	1.23	0.749	0.069	0.55 to 1.02	96	1.58	0.591	<0.001***	0.46 to 0.75	130	0.17	−0.132	0.007**	−0.23 to −0.04

Results of regression analysis examining the relationship between age groups and disease duration activity during follow-up per disease duration group. Rate ratios or beta coefficients, along with their corresponding 95% confidence intervals, illustrate the difference between each age group and the reference age group (25–30 years) for the number of contrast-enhancing lesions (CELs), number of new T2 lesions and the annualized relapse rate (ARR) across various disease duration subgroups.

ARR: annualized relapse rate; CELs: contrast-enhancing lesions; CI: confidence interval; RR: rate ratio; β: regression coefficient.

* *p* < 0.050; **p < 0.010; ***p < 0.001.

## Discussion

Our study confirms that older age is associated with a decrease in clinical and radiological inflammatory disease activity in a pooled clinical trial data set of people with RRMS. Moreover, we found that the association between age and inflammatory disease activity during follow-up was present in different subgroups based on disease duration.

A novel finding of our study is that the decrease in focal inflammatory disease activity seems to occur independent of disease duration; we saw a similar pattern of a decrease of relapses, CELs, and new T2 lesions on MRI also in patients with a relatively short disease duration. When repeating our analyses with age as a continuous factor, the results are comparable. Moreover, we found that there was no association between disease duration and inflammatory disease activity (data not shown) which supports that age, rather than disease duration, is the significant predictor of focal inflammation. Several studies on the association between age and inflammatory disease activity in people with MS have reported a significant association between age and clinical and radiological inflammatory disease activity. Relapse rates decrease in older people with RRMS and over time, and multiple studies have found an association between age and the presence of CELs on MRI.^1–4,16,17^ Our study adds to this that older RRMS patients also develop fewer new T2 lesions, a sensitive marker of focal inflammation in RRMS.

These findings inform clinical practice since people with RRMS currently start DMT relatively early in the disease course, and age is often not taken into account when making treatment decisions.^18^ Most current DMTs are aimed at preventing focal inflammation.^19^ Due to the natural decrease in focal inflammation in older people with MS, the relative added value of DMT also decreases in higher age. On the contrary, the risk of (infectious) side effects of DMTs increases in older people, and the increased prevalence of comorbidities, such as cardiovascular and metabolic diseases complicates treatment, often in association with a weakened immune system.^20,21^ The combination of lower inflammatory activity and lower tolerability makes for a different risk–benefit ratio in older patients compared to younger patients.

The observation that the association between age and focal inflammation is independent of disease duration suggests that physicians should consider age as an important factor in the disease course of the MS patient, as higher age was associated with a less inflammatory disease activity, even in patients with the shortest disease duration. It is noteworthy in this regard that several studies found that the risk of clinical and radiological inflammatory disease activity after discontinuation DMT decreases at higher age.^22–26^ A recently conducted RCT on discontinuation of DMT in people with MS aged ⩾55 years, median age of 63 years at enrollment, found that most people in the “discontinuation” group remained stable after DMT discontinuation, although the percentage of disease activity was higher in the “discontinuation” group than in the “continuation” group, and therefore, no definite conclusion on non-inferiority of DMT discontinuation could be made.^27^ Current treatment algorithms do not take into account the relationship between age and reduced clinical and MRI activity, and since inflammatory activity reduces with aging, it is likely that older patients require aggressive treatments less often than children or younger adults. However, patients should still be profiled for initiation of DMTs independently of age. The exact implication of lower inflammatory events in older patients needs to be studied in trials and observational studies that include patients with broader inclusion age ranges. Future studies will address the question of how to introduce age into the personalized prediction of treatment response and risk profile.

Our investigation has several limitations. First, we used clinical trial data sets for our analysis and all of the trials we used had specific entry criteria with regard to pre-trial disease activity and maximum age (56 years). The selective inclusion of people with more disease activity may have led to the inclusion of fewer older participants. However, this leads in fact to an underestimation of the effect of age. Another limitation is that despite the large sample size, participants with extreme presentations (younger people with long disease duration and older people with short disease durations) are still underrepresented. Clinical trials on DMTs in RRMS should include older patients to study the efficacy and side effects on DMTs in these specific age categories. Current knowledge of treatment efficacy is based on clinical trials in which people with a limited age range are included, mostly between 18 and 50 or 18 and 55 years. Post hoc analyses of these trials showed that the benefit of DMT was the highest in the younger age groups and decreases in the higher age groups.^5–7^ Although one might extrapolate that this association remains present in patients over the age of 55 years, this is yet unknown.

In conclusion, our study further strengthens the finding that higher age is associated with a decrease in clinical and radiological inflammatory disease activity in people with RRMS and adds that this association is independent of disease duration. These findings support a tailored approach in treatment strategy in people with MS of different ages and underline the importance of including a wider age range in clinical trials in RRMS.
